# The Wellbeing of Healthcare Workers during COVID-19 Era in Public Primary Health Facilities in Johannesburg, South Africa

**DOI:** 10.3390/ijerph21030372

**Published:** 2024-03-20

**Authors:** Glory Makhado, Busisiwe Ntuli, Lindiwe Zungu, Ntevhe Thovhogi, Peter Modupi Mphekgwana, Sogolo Lucky Lebelo, Sphiwe Madiba, Perpetua Modjadji

**Affiliations:** 1 Department of Public Health, School of Health Care Sciences, Sefako Makgatho Health Sciences University, Pretoria 0208, South Africa; 2 College of Graduate Studies, University of South Africa, Pretoria 0003, South Africa; 3 Non-Communicable Disease Research Unit, South African Medical Research Council, Cape Town 7505, South Africa; 4 Research Administration and Development, University of Limpopo, Polokwane 0727, South Africa; 5 Department of Life and Consumer Sciences, College of Agriculture and Environmental Sciences, University of South Africa, Johannesburg 1709, South Africa; 6 Faculty of Health Sciences, University of Limpopo, Polokwane 0700, South Africa

**Keywords:** wellbeing, healthcare workers, health facilities, COVID-19 era, South Africa

## Abstract

As the world grappled with the COVID-19 pandemic, healthcare workers (HCWs) continued to provide uninterrupted health care service delivery; therefore, this disproportionately affected their wellbeing. Our study explored the wellbeing of HCWs during the COVID-19 era in public health facilities in the City of Johannesburg, Gauteng province, South Africa. A qualitative study was conducted among twenty (20) HCWs through face-to-face in-depth interviews (IDIs) in the form of semi-structured interviews, audiotapes, and transcribed verbatim, and thematically analyzed with NVivo version 10. The findings showed that over half of HCWs (aged between 27 and 60 years) tested positive for COVID-19. Also, one third of HCWs’ family members tested positive while some died due to COVID-19 infection. Informed by the workers’ wellbeing framework, four themes emerged with fourteen sub-themes. Firstly, unsafe work environment was characterized by human resource related challenges such as increased workload; staff shortage; insufficient resources, e.g., personal protective equipment (PPE); poor policies in terms of compensation/allowance for being infected with COVID-19; poor health services; and death of colleagues. Secondly, poor health outcomes were described as strained emotional (psychosocial distress) and physical (respiratory related conditions) wellbeing. Thirdly, home and community environments were negatively impacted by interrupted relationships with family and friends, and experiences of deaths of loved ones. Finally, HCWs engaged personal wellbeing strategies through self-motivation; staying positive; family support; and participating in resilience-promoting extra mural activities to cope during the pandemic. In conclusion, the wellbeing of HCWs was aggravated during the COVID-19 era and led to low morale and compromised healthcare quality. This study advocates for promotion of greater resilience, and psychological and physical safety of HCWs through evidence-based, multilevel-multicomponent interventions at the workplace, home, and community environments in addition to strengthening public health policies and response to future pandemics.

## 1. Introduction

According to the Centers of Diseases Control and Prevention (CDC), the ability of individuals to address normal stresses, work productively, and realize one’s highest potential is considered as wellbeing [1,2]. Wellbeing refers to a state of complete physical, mental, and social wellbeing and not merely the absence of disease [3], but is also considered as the ability of individuals to address normal stresses, work productively, and realize one’s highest potential [1]. This concept of wellbeing remains an important occupational health aspect characterizing quality of life with respect to individuals’ health, and work-related environmental, organizational, and psychosocial factors [2]. The literature documents that workers in good health have higher odds to deliver optimal performance in the workplace [1,4,5].

On the other hand, the World Health Organization (WHO) has estimated a shortfall of approximately 18 million healthcare workers (HCWs), especially in low- and lower-middle income countries (LMICs), including countries whose socioeconomic status is affected by employment, deployment, retention, and performance of HCWs [6]. The wellbeing of HCWs influences the performance of organizations, workplace relationships, and quality sleep associated with high performance, patient satisfaction, lower turnover intentions, and increased levels of resilience [7]. However, HCWs are exposed to circumstances within their workplace, which negatively influence their physical, mental, and emotional wellbeing [8,9,10].

The emergence of COVID-19, also known as Acute Respiratory Syndrome Corona Virus-2 (SARS-CoV-2) was first reported in China (i.e., Wuhan) in 2019 [11,12,13] and spread throughout the globe. As of February 2020, the first case of COVID-19 was recorded in Africa (i.e., Egypt) [14]. Studies in LMICs reported the impact of COVID-19 on the physical and psychological wellbeing of HCWs, which informed recommendations mostly on mental health support among this workforce [15,16,17,18,19,20,21,22]. Prolonged exposure to COVID-19 at workplaces affected HCWs’ wellbeing [23,24] whilst they were expected to curb the spread of the virus without proper protection and guidelines [25,26]. WHO has so far raised concerns on the impact of COVID-19 pandemic on the wellbeing of HCWs [27].

South Africa was the most affected country in Africa, and had 4,076,463 confirmed cases, 102,595 deaths and 3,912,506 recovered cases [28,29,30]. The country endured lockdown, which imposed levels of restricted activities during the five waves of the pandemic [31], and were abolished later in June 2022 [32]. By the 27th of February 2023, approximately 38 million South Africans had received COVID-19 vaccines [33]. Many workplaces in South Africa had introduced mandatory vaccine policies [34] enshrined in policies under section 24(a) of the Constitution on mandatory vaccination and the right to environments conducive for wellbeing [35,36]. This is in alignment with the Occupational Health and Safety Act No. 85 of 1993 [37] and the operational requirements of the workplace in the country [38]. However, concerns are still looming around some future COVID-19 variants, which might pose further threat to individuals’ health because of their heightened virulence and pathogenicity inherent nature [39].

Prior to the COVID-19 era [30], HCWs were already strained psychologically and physically, while the health system of South Africa was already burdened with the convergence of non-communicable diseases (NCDs) such as diabetes, hypertension, and communicable diseases (CDs) such as HIV and TB [40,41]. The pandemic worsened the pre-existing challenges in health service delivery, especially in the public sector [42,43]. The challenges were further exacerbated by rapid increase in causalities, unfavorable work environment, and hospitalization [19,44,45,46]. Few qualitative studies have documented the wellbeing of HCWs in South Africa during the COVID-19 era [43,46,47,48,49]; however, there is minimal research in Africa. Gauteng province is one of the nine provinces of South Africa and is regarded as the economic hub in South Africa. The province has Johannesburg as its largest city and had experienced a rapid rise of COVID-19 infections [50,51].

In view of this, we conducted a descriptive qualitative study to explore the wellbeing of HCWs during the COVID-19 era in public health facilities in the City of Johannesburg, South Africa. Therefore, the main research question was “What was the wellbeing status of HCWs during the COVID-19 era in public health facilities in the City of Johannesburg?” The current study has the potential to inform contextual health promotion interventions for the wellbeing of HCWs with respect to their psychological health, workplace, home, and community environment, as well as effective current wellbeing strategies and preparations for future outbreaks.

## 2. Materials and Methods

### 2.1. Study Design

A descriptive qualitative design was used to explore the wellbeing of HCWs during the COVID-19 era in selected public health facilities in the City of Johannesburg, South Africa. The study adhered to the Consolidated Criteria for Reporting Qualitative Research (COREQ) [52].

### 2.2. Theoretical Framework

**Figure 1:**
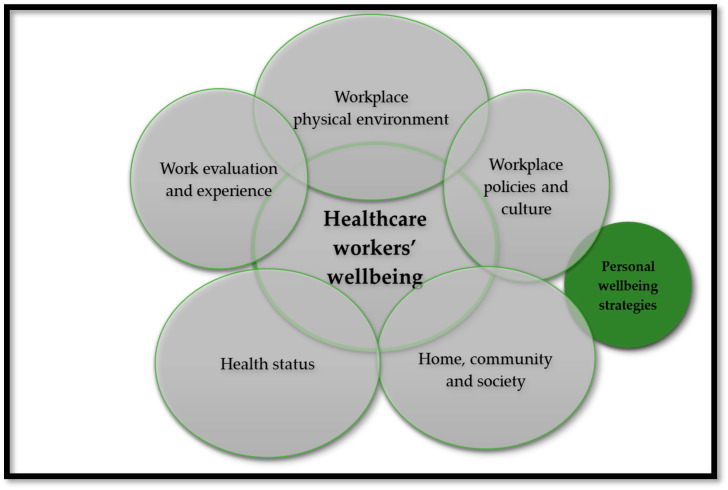
Adapted framework for workers’ wellbeing [55].

Previous studies have demonstrated that the wellbeing of workers was predicted by workplace conditions [53,54], including their health status and other environments such as home and community [55]. This study was informed by an adapted version of the workers’ wellbeing framework (see Figure 1), widely used to study wellbeing as an integrative concept that characterizes quality of life with respect to an individual’s health and work-related environmental, organizational, and psychosocial factors [55]. The framework comprises of five domains, namely, (1) workplace physical environment and safety climate; (2) workplace policies and culture; (3) health status; (4) work evaluation and experience; and (5) home, community, and society. COVID-19 featured in all the domains to explore the wellbeing of HCWs during the pandemic. For this study, we combined workplace information on environment, experience, and policies as “workplace environment”, while the “health status” stands on its own, as well as a “home and community environments”. The construct on “personal wellbeing strategies” in this study evolved during interviews when HCWs explained their capacity for managing personal functioning and adapting to the environmental demands while managing stressful situations [56,57,58,59]. This framework informed data collection and tools, as well as data analysis and interpretation of the findings.

### 2.3. Study Setting and Population

This study was conducted in the public primary health facilities situated in the City of Johannesburg Metropolitan Municipality (COJ). COJ forms part of Gauteng, and has an approximated population of 16.1 million people [60]. Gauteng province is bordered by four provinces, which are Mpumalanga, Limpopo, Free State and Northwest, and has experienced a rapid resurgence of COVID-19 infections [50,51]. There are 54 municipal facilities out of a total of 125 primary health facilities at COJ providing various services from maternal and child health care, chronic health, management of minor ailments to emergency care. The facilities operated from Monday to Friday from 7:30 am to 4:00 pm and Saturday from 7:30 am to 1:00 pm, and three to six HCWs were observed per facility. Various research studies from the Sefako Makgatho Health Sciences University (SMU) in South Africa have been conducted in COJ. These facilities have two different management teams: community health care centers (CHCs), classified with provincial clinics and managed by the Department of Health, whereas the municipal clinics and mobile clinics are under the management of the local governance of COJ.

### 2.4. Sampling and Recruitment Strategies

Purposive sampling providing in-depth and meaningful information about the topic of the study was used to select municipal facilities based on the highest head count of frontline HCWs and accessibility by a larger population. HCWs were identified and recruited through the support of the facilities managers. During recruitment, research team members explained the purpose of the study to individual participants as we approached them during their individual breaks. Those who agreed to participate in the study were requested to see the main researcher to arrange for individual in-depth-interviews (IDIs).

For this study, HCWs who were permanently employed with a working experience of at least 6 months at primary care level and had a direct contact with both suspected and confirmed COVID-19 patients were eligible to participate in the study. HCWs who fitted the criteria were willing and eager to participate in the study; as a result, obtaining consent for participation was not an issue in this study. The study excluded anyone who was providing healthcare at these facilities as a student or a volunteer, as well as HCWs who were not permanently employed.

### 2.5. Data Collections and Tools

**Figure 2:**
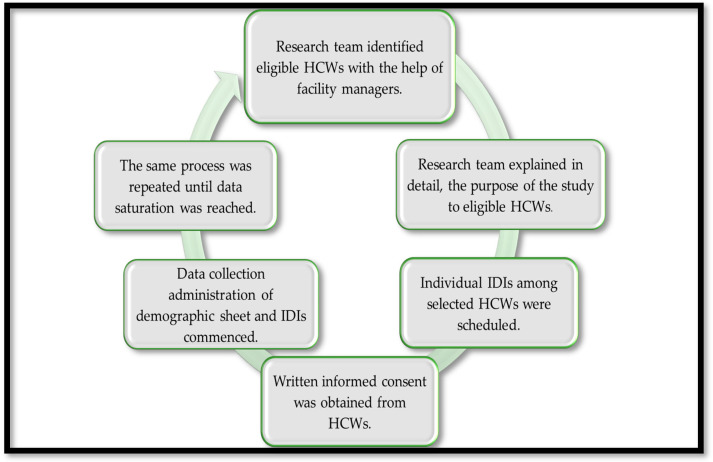
Schematic diagram of recruitment process and data collection. (HCWs stands for healthcare workers and IDIs stands for in-depth interviews).

Face-to-face IDIs were conducted using a semi-structured interview guide with open ended questions, informed by similar studies [7,43,46,47,61] between July 2022 and December 2022. The opening question on the interview guide addressed questions on how it felt being a HCWs during the COVID-19 era having to take a COVID-19 test and waiting for the results in general. Further questions were asked on workplace physical environment, workplace policies and culture; health status; work evaluation and experience; and home, community, and society. During interviews, responses were further explored, and clarity was sought through follow-up questions and probes. The recruitment process and data collection are schematically presented in Figure 2, below.

IDIs were conducted in a private room during breaks. HCWs were screened for COVID-19 before data were collected, although a few refused to be screened. We adhered to safety precautions of the COVID-19 pandemic by wearing personal protective equipment, maintaining hygiene practices and distancing during data collection. One HCW was used to pilot the consistency of a tool before the main study, and the instrument remained the same after pre-testing.

IDIs were conducted in English, and lasted for 30 to 60 min per interview, audio-recorded and transcribed verbatim (G.M.). Each participant was interviewed once, and neither transcripts nor findings were returned to participants for comment.

### 2.6. Data Management and Analysis

Thematic analysis was applied after recorded interviews were transcribed verbatim from the local language to English and uploaded on NVivo version 10. Transcripts were repeatedly read by authors to immerse themselves with data, manual coding and developing a codebook. Thereafter, codes, themes and subthemes were defined and refined, and illustrated the wellbeing of HCWs during the COVID-19 era. Concurrent data collection and analysis were conducted, and ended when saturation was reached in the 18th IDI and confirmed through 19th and 20th IDIs.

Use of a good digital recorder, recording interview notes, and coding were strategies used to ensure trustworthiness. After each IDI, we held peer debriefing sessions and triangulated data, and audit trail on data collection processes, analysis, and findings were stored safely. To avoid pre-conceived responses during interviews, bracketing was maintained, as well as during data analysis and interpretation to ensure minimal bias.

### 2.7. Ethical Considerations

This study received ethical clearance from the Sefako Makgatho Health Sciences University Research and Ethics Committee (SMUREC) (SMUREC/H/141/2022: PG). Permissions were obtained from the Research Committee of Johannesburg Health District in Gauteng Province, South Africa (Approval reference number: GP_202207_051). Further permissions were obtained from the managers of facilities situated in COJ municipality. Written informed consent was obtained from the HCWs for participation. Prior to the interviews, participants were informed that participation is voluntary, and pseudonyms will be used to maintain privacy and confidentiality.

## 3. Results

### 3.1. Characteristics of Participants

Most participants were females, aged below 40 years, single, with certificate/diploma qualifications, professional nurses, between 10 and 20 years of service, living in households with adults and children, and had monthly income of ≤R30,000 (<USD 1646). Over half of HCWs tested positive for COVID-19, while one third had multiple family members who had tested positive and one third had lost a close person due to COVID-19 infection. (Table 1).

### 3.2. Emergent Themes

**Figure 3:**
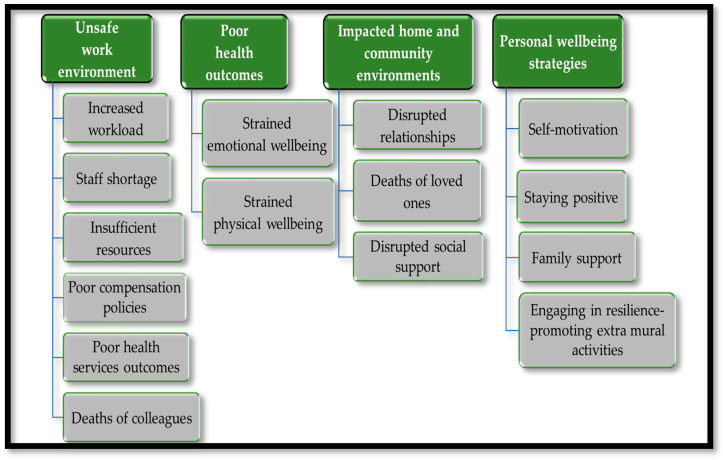
Summary of emergent themes.

Using the adapted workers’ wellbeing model, four themes with fourteen sub-themes emerged from the narratives of HCWs: (1) unsafe workplace environment (subthemes—increased workload; staff shortage; poor health services; death of colleagues; and low morale; (2) poor health outcomes (subthemes—strained emotional and physical wellbeing) (3) impacted home and community environments (subthemes—interruption of relationship with family and friends; and experiences of deaths of loved ones; and (4) (iv) engaged personal wellbeing strategies (subthemes—self-motivation; staying positive; family support; and participation in resilience-promoting extra mural activities), presented in Figure 3.

At the time of the study (July 2022–December 2022), South Africa had endured five waves of COVID-19 with lockdown restrictions reaching level five, restricting movement all together, and wearing face masks all the time, which were later abolished in June 2022 [31]. By then, over half of the participants in this study had tested positive for COVID-19. HCWs were interviewed face-to-face to explore their wellbeing during the COVID-19 era. The following themes from their IDIs categorized under four domains:

#### 3.2.1. Unsafe Workplace Environment

HCWs narrated unsafe work environment characterized by increased workload; staff shortage; insufficient personal protective equipment (PPE) and compensation allowance; poor health services outcomes; and death of colleagues. These issues came across as compromised work policies, which led to HCWs concerns of unsafety at work during COVID-19. Several issues mentioned above such as increased workload, staff shortage, and lack of support from the employer were also implicated.

▪ On increased workload, participants said:

‘*The workload was too much.*’

(40 years old, female, enrolled nurse, 14 years of work experience)

‘*The workload was too much, it was heavy…*’

(27 years old, female, professional nurse, 5 years of work experience)

‘*There was a time where a lot of staff were infected, and they didn’t come to work so it increased the workload to the ones that were left to work at the facility. It makes one to have a heavy workload because we must reallocate and must cover every service.*’

(50 years old, female, professional nurse, 24 years of work experience)

▪ On staff shortage, participants said:

‘*Shortage of staff was an issue, staff was sick.*’

(35 years old, female, enrolled nurse, 6 years of work experience)

‘*We were always short staffed, which meant those that were in the workplace now they must compensate for those who are absent.*’

(39 years old, female, medical doctor,14 years of work experience)

‘*I think the number one, shortage of staff…because we would never go for a week without one of us getting sick with COVID-19…*’

(37 years old, female, professional nurse, 17 years of work experience)

‘*Most of workers here had COVID-19 so it obviously had a strain because you find that maybe about four people or so are off sick or on quarantine for that seven or fourteen days so that obviously puts a lot of strain on ones that are on duty on that time because they must stretch to see all these patients.*’

(33 years old, female, professional nurse, 14 years of work experience)

▪ On deaths of colleagues, one participant said:

‘*We’ve lost colleagues to the pandemic… Therefore, we constantly felt unsafe…*’

(39 years old, female, medical doctor, 14 years of work experience)

▪ On insufficient resources and poor policies, one participant said,

‘*I think we were demotivated because at some point you would find that there is no PPE, it’s out of stock. And they didn’t want to give COVID-19 allowance, danger allowance and that is demotivating…*’

(27 years old, female, Professional Nurse, 5 years of work experience)

▪ On poor quality service, some HCWs indicated that increased workload, staff shortage and low morale translated into poor quality of service HCWs rendered to the patients. They said:

‘*We try and push harder. At times you feel that you are not giving proper nursing care… It was a disaster. There was a time that a patients consulted with me, and I realize that I will go to prison here because I was not offering good services. But what can you do, because at the end of the day all these patients must be seen.*’

(36 years old, female, professional nurse, 9 years of work experience)

‘*It was difficult because in my team I could see seventy patients a day. Already I was not providing quality service, I was doing quantity.*’

(36 years old, female, professional nurse, 11 years work experience)

‘*… Sometimes you are not rendering quality service to the patients because you get exhausted, there was no quality of care*’

(40 years old, female, professional nurse, 5 years of work experience)

Collectively, the wellbeing of HCWs was negatively aggravated by the above-mentioned sub-themes, and collectively led to their low morale, as well as potentially compromised the quality of healthcare service during the COVID-19 era. Most of the participants said:

‘*Staff morale was low, because there was too much workload and most of the time, we had shortage of staff because part of our staff had to go in isolation.*’

(30 years old, female, professional nurse, 7 years of work experience)

‘*The mood was very low. People were not motivated.*’

(39 years old, female, medical doctor, 14 years of work experience)

‘*You get demotivated, your morale goes down, you lack ability to wake up every morning… Your colleagues are sick, you are even afraid that maybe next is me…*’

(37 years old, male, professional nurse, 10 years of work experience)

‘*…we were demotivated because at some point you would find that there is no Personal Protective Equipment, it’s out of stock. And they didn’t want to give us COVID-19 pandemic allowance, danger allowance and that is demotivating. Your morale will be low.*’

(27 years old, female, professional nurse, 5 years of work experience)

‘*… Already I was not providing quality service, I was doing quantity.*’

(36 years old, female, professional nurse, 11 years work experience)

#### 3.2.2. Poor Health Outcomes

Regarding poor health outcomes, HCWs reported that their emotional and physical wellbeing were strained. Emotionally, they experienced psychological distresses of fear of dying, stress, and mental exhaustion, accompanied by emotional roller coaster of sadness, depression, anxiety, temper, frustration, worriedness, insomnia, and trauma. While, physically, they reported fever, asthma, recurring bronchitis, fatigue, and unending physical exhaustion.

▪ On emotional wellbeing, some participants said:

‘*The situation was emotional unstable, next again, stable, and then unstable. One minute you are over thinking, one minute you are fine, one minute you are scared, one minute you are okay.*’

(34 years old, female, professional nurse, 10 years of work experience)

‘*I was feeling anxious as well because we didn’t know this pandemic. I was anxious…*’

(37 years old, male, professional nurse, 10 years of work experience)

‘*There were times I wanted to call the hotline for psychiatric because I felt like I was feeling sad and depressed. Waking up was a mission, asking myself if I really want to go there, especially if you are working at the COVID-19 tent because everybody who is coughing will be coming to you.*’

(28 years old, female, professional nurse, 10 years work experience)

‘*I was short tempered, … I was frustrated, you don’t know what’s going to happen.*’

(36 years old, female, professional nurse, 9 years of work experience)

‘*I used to worry a lot, like what if I get infected and I don’t survive…*’

(40 years old, female, enrolled nurse, 14 years of work experience)

‘*… I ended up drinking sleeping pills so that I can sleep for me to forget what was happening, … I couldn’t sleep…*’

(35 years old, female, enrolled nurse, 6 years of work experience)

‘*You don’t understand, I was always traumatized… It affected me in so many ways, and I don’t want to go back there*’

(40 years old, female, enrolled nurse, 14 years of work experience)

Emotional wellbeing was described as stress during the COVID-19 pandemic trying not to get infected, while at the same time providing health service. Participants said:

‘*It was a very stressful time. Very, very stressful. I ended up being admitted at a psychiatric hospital because of stress you know. You know you get stressed.*’

(60 years old, female, professional nurse, 37 years of work experience)

‘*It’s been a stressful moment because every time you feel like you are trying to avoid being infected with COVID-19, at the same time you try to provide service as expected, and that is exhausting.*’

(33 years old, female, professional nurse,14 years of work experience)

‘*It was a time that was quite stressful for all of us. It was something that we have never experienced.*’

(39 years old, female, medical doctor,14 years of work experience)

Fear for their lives, being infected, hospitalized and/or dying were other points HCWs raised that affected their emotional wellbeing. Some said:

‘*…We were fearing for our health …*’

(60 years old, female, professional nurse, 37 years of work experience)

‘*It was hectic, fearing for our lives. People were dying, and you are thinking am I going to die. There was a time where I personally was infected with COVID-19, and I thought I am dying.*’

(36 years old, female, professional nurse, 9 years of work experience)

‘*I was afraid of dying due to COVID-19 infection.*’

(40 years old, female, Professional Nurse, 5 years of work experience)

‘*It was just scary because every day you hear that someone somewhere has passed away, you become scared, am I going to make it end of the week? end of the month? You become scared.*’

(37 years old, female, professional nurse, 17 years of work experience)

‘*…I was scared to get COVID-19 and complicate, get hospitalized and even die.*’

(39 years old, female, professional nurse, 12 years of work experience)

Further HCWs’ expressions entailed mental exhaustion, which led to a feeling of not wanting to come to work. Participants said:

‘*My mental exhaustion was all over the place… My mind will tell me that if I go home what if I am taking COVID-19 home, what if I give my family COVID-19…*’

(27 years old, female, professional nurse, 5 years of work experience)

‘*…I ended up drinking sleeping pills so that I can sleep for me to forget what was happening, so I was not okay mentally… My mind was not well*’

(35 years old, female, enrolled nurse, 6 years of work experience)

‘*I got exhausted number one, so once you get exhausted it means emotionally you are not okay, so you find that even when you get home you don’t have time to do things that are personal to you because you are exhausted emotionally… Social life was just on the low because we were always tired*’

(33 years old, female, professional nurse,14 years of work experience)

‘*Just tiredness, like mentally I can say I am tired*’

(39 years old, female, enrolled nursing assistant, 9 years of work experience)

‘*It really exhausted me emotionally. Sometimes when I wake up in the morning, I snooze the alarm hundred times thinking oh my God I am going to that place*’

(35 years old, female, Enrolled Nurse, 6 years of work experience)

▪ On physical wellbeing, HCWs reported suffering from fever, asthma, recurring bronchitis, fatigue, and unending physical exhaustion. This is what some of them said:

‘*When you get home, you feel so tired, … physically, I just feel exhausted.*’

(30 years old, female, professional nurse, 7 years of work experience)

‘*I am still exhausted, but I think the exhaustion is more from the COVID-19 pandemic more than anything. Since then, I am just physically exhausted, I come to work and go home but I can feel I am exhausted.*’

(36 years old, female, professional nurse, 9 years of work experience)

‘*Sometimes you end up shouting at patients only because you are physically exhausted, which I have been more since COVID-19.*’

(40 years old, female, enrolled nurse, 14 years of work experience)

‘*Remember there are some of us who had COVID-19 and recovered, but we got the post COVID syndrome. We don’t have our full pre COVID physical health. Some of us are getting recurrent bronchitis, others are now borderline asthma, others are weak. Others have diabetes because of their COVID-19 infection.*’

(39 years old, female, medical doctor, 14 years of work experience)

‘*There are those colleagues who were directly infected by COVID-19, they will tell you even today some of them are still having post COVID-19 fatigue. Some of them will tell you that ever since I had COVID-19 it’s like my immune system is no longer the same, I feel like I get feverish more often, my body is no longer physically strong as before.*’

(30 years old, female, professional nurse, 7 years of work experience)

#### 3.2.3. Impacted Home and Community Environments

HCWs narrated impacted home and community environments manifested through interruption of relationships with family and friends, and experiences of deaths of loved ones. This was due to social distancing and quarantine from family members and friends because of fear to infect their loved ones with COVID-19, while some of the loved ones died due to COVID-19 infection.

▪ On social distancing and quarantine, they said:

‘*Not being able to go to your children and husband is draining while you are sick, and you don’t have anyone to take care of you, only yourself… My husband had COVID-19, he moved out of the house and left the kids, so I had to look for someone to come and take care of the kids because I am this side working and not staying. with them.*’

(40 years old, female, enrolled nurse, 14 years of work experience)

‘*I had to be separated from my son during COVID-19 pandemic because he couldn’t go to school so that for me was just not right because I was used to staying with him every day, I had to take him home to granny.*’

(33 years old, female, Professional Nurse,14 years of work experience)

‘*The condition at home changed, we were talking over the phone with my siblings, there were no visits because I am a health care worker, so socially we were affected. I remember we could not even do a birthday party for my mom and my siblings had to video call her on her birthday. They were afraid because they said I am a nurse I am going to give them COVID-19. Most of the things were done via the phone…*’

(49 years old, female, professional nurse, 12 years work experience)

‘*My partner was pregnant at that time of COVID-19. So, we were sleeping in two separate bedrooms trying to protect her, and the relationship was affected.*’

(47 years old, male, professional nurse,15 years work experience)

▪ On losing family and friends, they said:

‘*I lost my mother.*’ (*Participant burst into tears*)

(49 years old, female, professional nurse, 12 years work experience)

‘*I lost my father, uncle and a colleague.*’ (*Participant had teary eyes*)

(35 years old, female, enrolled nurse, 6 years of work experience)

‘*I lost nine cousins and one nephew within a space of two months.*’

(60 years old, female, professional nurse, 37 years of work experience)

‘*…some of my friends died of COVID-19…*’

(35 years old, female, enrolled nurse, 6 years of work experience)

‘*…we’ve lost family members. We’ve lost friends… I lost my aunt and cousin*’

(39 years old, female, medical doctor, 14 years of work experience)

‘*I know a colleague of mine who lost a mother, who lost a grandmother to COVID-19 so you can understand she lost her support system.*’

(30 years old, female, professional nurse, 7 years of work experience)

#### 3.2.4. Engaging Personal Wellbeing Strategies

Lastly, older HCWs (>30 years) with longer work experience (7 to 14 years) alluded to having engaged personal wellbeing strategies through self-motivation; staying positive; family support; and participation in resilience-promoting extra mural activities to cope during the pandemic.

▪ On staying positive, they said: ‘*… I just knew that I must stay positive despite…*’ (37 years old, male, professional nurse, 10 years of work experience).

‘*There was a time where I even stopped reading news because all I could see was how many people were dying, how many people were hospitalized so I had to shut that down…and think positive. That’s how I coped.*’

(30 years old, female, professional nurse, 7 years of work experience)

‘*…I have my personal motivation so that one can wake up every day and go to work and still come back and be with family…*’

(39 years old, female, medical doctor, 14 years of work experience)

▪ Family support was also highlighted by some HCWs, and they said: ‘*I had support from my family*, *they were there for me*.’ (37 years old, male, professional nurse, 10 years of work experience)

Another HCWs said ‘*I know a colleague of mine who lost a mother, who lost a grandmother to COVID-19 so you can understand she lost her support system.*’

(30 years old, female, professional nurse, 7 years of work experience)

▪ On extra mural activities, one said: ‘*I tried to plan time for rest, and I used the gym to try and offload. After work I go to the gym and I also try to sleep early, every day by eight to nine in the night I am in bed.*’ (34 years old, female, professional nurse, 10 years of work experience)

The other one said: ‘*One dig deeper and reach out to faith in God and engage with pastors… So*, *I think prayer life and my faith really helped me*.’ (39 years old, female, medical doctor, 14 years of work experience).

## 4. Discussion

The alarming context of the COVID-19 health emergency happened when there were no protocols and guidelines for HCWs to provide care to patients infected with COVID-19, worldwide, including South Africa. Therefore, countries were unprepared to deal with the pandemic [50,62] since there were uncertainties regarding the treatment of COVID-19, except for promising alternative treatments [63]. Amidst these concerns, the wellbeing of HCWs during the pandemic was negatively impacted through unsafe work environment; poor health outcomes; and home and community environments. These led to HWCs being resilient and seeking coping strategies to deal with the burden of the pandemic while they continued to provide healthcare services under the unique challenges of an outbreak [3,13,26,61]. This is supported by the literature that in cases such as this, HCWs have tendencies to employ strategies that protect their wellbeing and engage in resilience-promoting interventions [56,59].

Over half of the HCWs in this study, tested positive for COVID-19 and this was consistent with studies that reported unprecedented number of HCWs infected with COVID-19 during the pandemic in South Africa [64,65]. Approximately 16,236 HCWs have been infected with COVID-19 since the beginning of the pandemic in 2020, of whom 1908 were hospitalized, and over 100 died. In November 2021, roughly 55,109 (64%) HCWs in Gauteng were fully vaccinated against COVID-19, while at least 36% were not vaccinated, despite being frontliners during the pandemic [66]. This study further showed that a third of HCWs refused to vaccinate for COVID-19, despite being faced with a rapid influx of critically unwell patients and having to protect themselves from being infected [25,26,67]. Unbeknownst to us in this study, since the issue of vaccination was only part of the demographics with close ended questions, we could deduce from literature that refusal to vaccinate might have been due to misinformation, lack of knowledge, mistrust in experts and authorities, and competition amongst pharmaceutical companies, vaccine safety, side effects, and efficacy [68]. Furthermore, one third of HCWs’ family members tested positive while some died due to COVID-19 infection in this study. COVID-19 infected and affected HCWs during the pandemic while several of them experienced the death of loved ones due to the infection [69].

Regarding unsafe workplace environment, human resource challenges reported in this study were increased workload, staff shortages, insufficient resources such as personal protective equipment (PPE), and poor policies in terms of compensation COVID-19 allowance, as well as poor health services, and deaths of colleagues. Concerns on insufficient supply of PPE, and lack of compensation allowance, which potentially affected quality of service were highlighted among HCWs in this study. It is documented that resources, such as shortages of staff, and shortage of PPE were limited in healthcare environment during COVID-19, and negatively affected the capacity of HCWs to provide appropriate patient care and affected their wellbeing [70,71], as reported in a scoping review [25]. A similar situation was also reported in South Africa during the pandemic, burdening the health system [72], and increased staff turnover intention [73,74,75,76,77]. Apparently, most of their colleagues were sick and on leave or quarantining during the pandemic. Therefore, workload, irregular work routines and job dissatisfaction due to the indispositions of the COVID-19 pandemic are some of the issues that affected the wellbeing of HCWs [74,75,76,77]. Shortage of staff and the scarcity of PPE were some of the major concerns and challenges among HCWs and might have potentially affected their wellbeing and quality of service rendered.

Concerning poor health outcomes among HCWs in this study, their emotional and physical wellbeing were strained. HCWs experienced psychological distresses in a form of fear of dying, mental exhaustion, accompanied by emotional roller coaster of sadness, depression, anxiety, temper, frustration, insomnia, and trauma. Additionally, HCWs also experienced fever, asthma, recurring bronchitis, fatigue, and unending physical exhaustion, similar to other local [43,46,47] and international studies [15,20,61]. Substantial pressure, fear of being infected, infecting others and dying have been reported among HCWS during the COVID-19 pandemic [77,78,79]. These fears have been implicated in psychological distress and perceived inability to cope effectively and discomfort [61,80] as reported in other countries such as in Saudi Arabia [81] and USA [82]. Psychological distress has been shown to be prevalent among HCWs [10,19] with increase rates of anxiety, depression, stress, irritability, insomnia, anger, and frustration [19,83,84,85]. Apparently, these were exacerbated by working in high-risk COVID-19 wards, with fear of exposure to the virus [22,86], leading to poor quality of care [71]. Therefore, provision of psychological support to frontline HCWs during pandemics remains paramount.

In the same vein, HCWs reported moral and post-traumatic stress injuries in this study such as unending physical exhaustion, feeling feverish, recurring bronchitis, asthma, weakened immune system, and persisting fatigue. This is consistent with studies in Asia, Europe, and USA [25,87,88] and in South Africa [89,90]. Moral injury is a form of psychological response, and in this case it refers to transgression from moral values when one feels that an institution has failed to provide care as expected [91,92], and it is associated with negative mental health outcomes, such as post-traumatic stress (PTS) [92,93]. PTS doubled among almost half of the HCWs during the second wave of COVID-19 pandemic in South Africa compared to a year prior [29]. Similar results were reported in China [94], Spain [95], while in some instances, lower levels of PTS have been reported in Singapore and India [83]. Caution should be taken during interpretations of PST among HCWs due to different time periods of the pandemic in the respective countries [96]. Nonetheless, evidently, HCWs in South Africa experienced higher levels of PTS than their counterparts in other countries [96], which seems to be the case in this study.

Impacted homes and community environments manifested as interrupted of relationships with family and friends, and experiences of deaths of loved ones. COVID-19 pandemic negatively affected the families of the HCWs beyond workplace, with loss of close persons and relatives. During the pandemic, HCWs had to prioritize their work over parenting, spending time with family and friends, and postponed their plans. HCWs spent many extra hours at work, leaving no time for their families, children, or partners, and found it difficult to cope during the pandemic. While work-family balance is a common challenge for HCWs due to work-shifts, this crisis came with unprecedented threats, and their spouses lived in a state of fear because of being aware of unfavorable work conditions, especially regarding shortage of staff and insufficient supply of PPE [97]. HCWs were always concerned about infecting close people with COVID-19 during the pandemic, hence, they were forced to stay far from their families [10,11,12,13]. This interrupted relationships, family support and coping [14]. This is because HCWs were in regular direct contact with patients, predisposed their family members to increased risk of contracting COVID-19 [70,98]. Therefore, social distancing, social isolation and quarantine were used as preventative measure recommended to flatten the curve of COVID-19, condoning solitude [77]. Similar to our study, these measures disrupted relationships with family and friends and exacerbated fears of infecting others and pain of losing close people during the pandemic.

Finally, HCWs in this study engaged personal wellbeing strategies through self-motivation; staying positive; family support; and participating in resilience-promoting extra mural activities to cope during the pandemic. Older HCWs with longer work experience in this study demonstrated resilience and coping. Literature documents that older adults appear to be coping better with COVID-19 than younger adults [99,100]. The difference in resilience and coping between younger and older HCWs could be because younger HCWs have a high risk of stress and health anxiety, since they have less experience in the workplace. However, there are inconsistent reports that age of HCWs does not determine coping mechanisms adopted [101] due to reasons such as equally fearing the disease, no misconceptions about the disease or incapable coping and lack of proper treatment [102]. Psychological support and debriefing intervention sessions focusing on suitable coping strategies to actively address stress among HCWs are also important [96]. HCWs use a range of techniques from religious/spiritual, social, and psychological approaches as coping strategies [57,103,104] reported in South Africa [57]. Lockdowns in South Africa also affected faith-based practices [56], because mass gatherings were prohibited. Furthermore, during the pandemic, social support seemed to be helpful for HCWs to maintain healthy emotional states [105], although made impossible by social distancing, lockdown and quarantine measures to connect with their families and friends [105].

### Limitations

The findings of this study should be considered with some limitations. Firstly, a small sample size of HCWs was achieved in this study, informed by data saturation reached at participant number 18 and ensured with additional two IDIs for participants 19 and 20. Secondly, recruitment through facility managers may have introduced some form of sampling bias since HCWs might have felt obligated to participate in the study. However, during recruitment, procedure and ethical considerations were clearly communicated, especially on autonomy with the basis on informed consent, truth-telling, and confidentiality. Thirdly, most HCWs who participated in this study were nurses; therefore, the findings cannot be generalized to other professionals and must be used with caution in other settings. Fourthly, coping strategies emerged during interviews, as a result the study did not initially consider application of standardized tools to study coping strategies of HCWs in detail. Use of a detailed tool for coping strategies in future studies might yield more information compared to the limited information reported in this study. The strength of this study relied on the fact that IDIs were conducted immediately after lockdown restrictions were abolished, while the memories of HCWs were still fresh to recall the impacts of the pandemic. Nonetheless, potential recall bias during interviews is acknowledged and HCWs were able to reflect on their wellbeing during the COVID-19 era.

## 5. Conclusions

The study suggested that the wellbeing of HCWs in the primary public health facilities of Johannesburg in South Africa was aggravated during the COVID-19 era and led to low morale and compromised the quality of healthcare service. This was firstly, through unsafe work environments that were characterized by human resource related challenges such as increased workload; staff shortage; insufficient resources such as PPE; poor policies in terms of compensation COVID-19 allowance; poor health services; and death of colleagues. Secondly, poor health outcomes were described as strained emotional (psycho-social distress) and physical (respiratory related conditions) wellbeing. Thirdly, home and community environments were negatively impacted by interrupted relationships with family and friends and experiences of deaths of loved ones. Finally, HCWs engaged personal wellbeing strategies through self-motivation; staying positive; family support; and participating in resilience-promoting extra mural activities to cope during the pandemic.

In conclusion, the wellbeing of HCWs was aggravated during the COVID-19 era and led to low morale and compromised healthcare quality. This study advocates for promotion of greater resilience, and psychological and physical safety of HCWs through evidence-based multilevel-multicomponent interventions at workplace, home, and community environments in addition to strengthening public health policies and responses to future pandemics. Also, future longitudinal studies are necessary to continuously assess the wellbeing of HCWs to inform timeous intervention.

## Figures and Tables

**Table 1:** Characteristics of participants.

Variables	Categories	*n* (%)
Sex	Male	2 (10)
	Females	18 (90)
Age (years)	<30	17 (85)
	≥30	3 (15)
Marital status	Single	11 (55)
	Married	9 (45)
Qualification level	Certificate/Diploma	12 (60)
	Degree/Postgraduate	8 (40)
Occupation	Professional Nurse	15 (75)
	Other HCWs	5 (25)
Duration of service (years)	<10	6 (30)
	10–20	12 (60)
	≥20	2 (10)
Monthly household income	≤R30,000 (<USD 1646)	11 (55)
	>R30,000 (≥USD 1646)	9 (45)
Household head	No	9 (45)
	Yes	11 (55)
Number of adults in a house	≤2	15 (75)
	>2	5 (25)
Number of children in a house	≤2	13 (65)
	>2	7 (35)
COVID-19 status	Tested negative	8 (44)
	Tested positive	10 (56)
Family member(s) tested positive for COVID-19	No	13 (65)
	Yes	7 (35)
Who (i.e., family member) tested positive	Spouse	5 (20)
	Child	5 (20)
	Parent/aunt/uncle	6 (24)
	sibling	9 (36)
Lost close person(s) due to COVID-19 infection	No	13 (65)
	Yes	7 (35)

*n*—stands for frequency, % for percentage, ZAR for South African Rand, and USD for United States Dollar.

## Data Availability

The data that support the findings of this study are available from the corresponding author, upon reasonable request.
